# Genome-wide and local pattern of linkage disequilibrium and persistence of phase for 3 Danish pig breeds

**DOI:** 10.1186/1471-2156-14-115

**Published:** 2013-12-05

**Authors:** Lei Wang, Peter Sørensen, Luc Janss, Tage Ostersen, David Edwards

**Affiliations:** 1 Department of Molecular Biology and Genetics, Aarhus University, Blichers Allé 20, 8830 Tjele, Denmark; 2 Breeding and Genetics, Pig Research Centre, Danish Agriculture & Food Council, Axeltorv 3, 1609 Copenhagen V, Denmark

**Keywords:** Linkage disequilibrium, Persistence of phase, Danish pig breeds, Model local LD, Visualize local LD variation

## Abstract

**Background:**

The extent of linkage disequilibrium (LD) is of critical importance for genomic selection and marker assisted selection. The primary purpose of this study is to examine patterns of LD in three Danish pig breeds (Duroc, Landrace and Yorkshire); we also examine patterns of persistence of phase between the breeds. We quantify local LD by fitting a model relating LD to physical distance between markers in sliding windows, and use this to visualize how LD varies according to physical position. We use a similar method to examine local persistence of phase.

**Results:**

Average LD decay over distance for Duroc was significantly different from Landrace and Yorkshire, that showed similar patterns. Persistence of phase between Landrace and Yorkshire was much higher than between these breeds and Duroc. Local *r*^2^ over the chromosomes showed more variation between breeds than average *r*^2^ decay across whole genome. Also local persistence of phase was higher between Landrace and Yorkshire than between these breeds and Duroc.

**Conclusions:**

The results concerning genome-wide LD indicated that Duroc had “old inbreeding”, and confirmed the mixture history of Landrace and Yorkshire, which is also implied by the higher level of persistence of phase between Landrace and Yorkshire. The method to estimate and visualize local pattern of LD and persistence of phase provides insight into how these quantities vary along chromosomes and between breeds.

## Background

Linkage disequilibrium (LD) measures the degree of non-random association of alleles at different loci in a population. Many recent research studies in both humans and animals [1-3] have focused on characterizing the patterns of LD. The extent of LD can reveal population history and breed development, and is related to effective population size [4,5]. Genomic selection in livestock relies on the existence of LD between causative variants and genetic markers [6]. Moreover, gene mapping of complex diseases or trait loci through genome-wide association studies (GWAS) depends crucially on the pattern of LD in the genome [7].

LD expresses the strength of allelic association within a population, whereas persistence of LD phase measures the degree of consistency of LD phase between two populations. Persistence of phase is important for several reasons. For example, it may explain why linkage between a marker and a QTL detected in one population cannot be confirmed in a second population [2]. Thus, persistence of phase influences the reliability of genomic prediction in multiple breeds. In addition, the range of distances for which persistence of phase between two populations extends can be used to determine marker density for a fine-mapping experiment, GWAS or genomic selection [2,8].

Many studies on LD in both human and livestock populations have been published recently [2-4,9]. These mainly relate the average decay of LD and persistence of phase to physical distance over the whole genome, and compare LD and persistence of phase between populations. However, it is known that recombination rates vary along the genome [10] and that selection and genetic drift can cause local genomic differences in inbreeding and effective population size [11]. It is therefore to be expected that LD and persistence of phase vary locally. Only a few studies investigate local patterns of LD [12], and to our knowledge no studies investigate local LD decay and local patterns of persistence of phase. More detailed knowledge on the local patterns of LD and persistence of phase may be useful for designing marker panels for genomic prediction across populations, or investigating selection effect on different chromosomal segments. To this end we develop a new method to visualize local patterns of LD and persistence of phase along the chromosomes, that do not require knowledge of recombination rate or effective population size.

The LD pattern and persistence of phase in Duroc, Landrace, and Yorkshire breeds in US and Finland have been investigated [3,4], but not in Danish breeds. The objective of this study is to investigate the patterns of LD and persistence of phase, within each breed and across breeds, on both genome-wide and local levels. For purposes of comparison we estimate and describe the average LD and persistence of phase for a certain distance range in the same way as other studies, and compare the average decay of LD and persistence of phase against distance between three breeds. We also apply our new visualization method to compare the differences of local LD and persistence of phase across breeds.

## Methods

### Genotyping data

For this study, the numbers of genotyped animals were 4249, 1979 and 2123 for Duroc, Landrace, and Yorkshire, and all animals were genotyped with the Illumina PorcineSNP60 Genotyping BeadChip (Illumina Inc.) [13]. If one animal had missing genotypes in more than 10% of the SNPs, it was excluded from the sample. Similarly, SNPs with genotypes available for less than 90% of the sample were removed from further analysis (*CallRate* < 0.9). In this study, only autosomal SNPs without uncertainty of position in assembly (Sscrofa10.2) were considered [14]. Markers with minor allele frequency (MAF) below 0.05 were also removed. Moreover, SNPs were excluded if departures from Hardy Weinberg equilibrium (*P* < 10^-7^) within breeds were observed. Additionally, minimum geneCall score per SNP per animal to be approved was 0.6. After applying these filtering criteria, a total of 29,567, 33,719 and 33,907 SNPs for Duroc, Landrace and Yorkshire breeds respectively were included in the analysis.

The animals from each breed were selected to be included in the analysis using the same strategy: first parents were excluded, and then one animal was randomly selected out of its full sibs. Therefore the population structures between samples for three breeds are quite similar, and family structure has been removed. So the analysis in this study were based on the current sample sizes of 2580, 902 and 889 for Duroc, Landrace and Yorkshire, respectively.

The present study was not subject to ethical approval since it was based on pre-existing data belonging to the Danish Agriculture and Food Council, Pig Research Centre, and so did not require the application of additional experimental procedures.

### Estimation of LD and persistence of phase

To estimate the extent of LD, we calculated the squared correlation coefficient between two loci (*r*^2^), which is a considerably more robust LD measure than *D*^′^. It is known that *r*^2^ is less dependent than *D*^′^ on allele frequencies [15], and so is more suitable for comparison of population-wise LD between breeds. We used Beagle [16] to impute missing genotypes and to construct haplotypes. Pairwise *r*^2^ was estimated according to the following equation [3]:

(1) $$r_{\text{ij}}^{2}=\frac{{\left(\;p_{\text{ij}}-p_{i}p_{j}\right)}^{2}}{p_{i}(1-p_{i})p_{j}(1-p_{j})},$$

where *p*_*i*_, *p*_*j*_ are the marginal allelic frequencies of marker *i* and *j* respectively, and *p*_*ij*_ is the haplotype frequency of these two markers. We used R [17] for all statistical computations.

As persistence of phase measures the consistency of LD phase between two populations, the correlation between *r*_*ij*(*A*)_ and *r*_*ij*(*B*)_ for a common set of markers between two populations *A* and *B* is calculated as [3]:

(2) $$\text{cor}(r_{A,B})=\frac{\underset{(i,j)\in p}{\sum }(r_{\text{ij}(A)}-{\bar{r}}_{A})(r_{\text{ij}(B)}-{\bar{r}}_{B})}{S_{A}S_{B}}$$

where *cor*(*r*_*A*,*B*_) is the correlation of phase between *r*_*ij*(*A*)_ in population A and *r*_*ij*(*B*)_ in population B, *S*_*A*_ and *S*_*B*_ are the standard deviation of *r*_*ij*(*A*)_ and *r*_*ij*(*B*)_ respectively, and ${\bar{r}}_{A}/{\bar{r}}_{B}$ are the average *r*_*ij*_ across all SNP *i* and *j* within the common set of markers.

In our study, we investigated the persistence of phase in two scenarios; in Scenario I, persistence of phase was estimated based on the common SNPs across all three breeds, and in Scenario II it was based on the common SNPs between each breed pair. The number of common SNPs used to estimate persistence of phase for all three breeds was 17,918 in Scenario I; in Scenario II, the numbers were 22,142 for Duroc-Landrace, 22,347 for Duroc-Yorkshire, and 26,505 for Landrace-Yorkshire. The reason that we included Scenario II was to study whether persistence of phase as estimated in Scenario I was altered when more markers were included.

In this paper we study two population features (LD for each breed, and persistence of phase between pair breeds) from two perspectives (genome-wide average decay over distance, and fine-scale “local” patterns along chromosome). So the methods and results present both LD and persistence of phase first at the genome-wide level, and then at the local level.

### Genome-wide average LD and persistence of phase

To investigate the pattern of genome-wide average LD and persistence of phase against the physical distance between markers, we first pool data from all autosomal chromosomes for each breed, split all marker pairs from autosomal chromosomes into groups according to physical distances between pair loci, then take the average value of *r*^2^ within each group. The groups were even intervals of 100 kb length, from 0 up to 10 Mb. For example, all the marker pairs $r_{\text{ij}}^{2}$ with distance less than 100 kb were in the first group, and the average value of *r*^2^ for these pairs was calculated. To estimate persistence of phase, we first grouped the SNP pairs according to their physical distances in the same way as for average LD decay. Then within each group, we calculated the correlation of *r*_*ij*_ in breed *A* with *r*_*ij*_ in breed *B* by Equation 2.

### Local LD map

Under the assumption of an isolated population with random mating, Sved (1971) [18] derived the equation of relationship between genetic distance (*c*, recombination rate), LD (*r*^2^) and effective population size (*N*_*e*_):

(3) $$E(r^{2})=1/(1+4{\text{cN}}_{e}),$$

Since the data under analysis have weak or no family structure, we are unable to obtain accurate genetic distances. We propose here to model the relationship between *r*^2^ and physical distance *d* using the same functional form as in Equation 3. This can be viewed as combining the local relationship between genetic and physical distance, and the local effective population size, into one parameter, *α*, in the following Equation 4 below. The model is a generalized linear model with Gamma distribution and inverse link, with expectation:

(4) $$E(r_{\text{ij}}^{2})=1/(1+\alpha d_{\text{ij}}),$$

where *d*_*ij*_ is the physical distance in Mb and *α* is the regression parameter to be estimated. When fitting the model, marker pairs with *r*^2^ = 0 are excluded. *α* reflects the rate of decay of *r*^2^ over distance, so that large values of *α* indicate a low extent of LD. Thus *α* can be interpreted in terms of “local” recombination rate and effective population size, as we discuss below. The reason for choosing Gamma distribution is that we observed an approximately quadratic relationship of mean to variance of *r*^2^, which is consistent with the assumption that the conditional distribution of *r*^2^ given *d* has a Gamma distribution.

We constructed local LD maps as follows. First, we used a sliding window technique including *N* markers in each window, and at each step moved one marker to the next window. For each window, we used all pairs of $r_{\text{ij}}^{2}$ and *d*_*ij*_ among these *N* markers to estimate ${\hat{\alpha }}_{\text{local}}$ using the above model. So a set of values ${\hat{\alpha }}_{\text{local}}$ was obtained for all windows. We then substituted $\hat{\alpha }$ and a moderate distance *d* in Equation 4 to obtain expected *r*^2^ between the marker at the middle point in the window with other markers of distance *d*. Finally we plotted these *r*^2^ against the physical position of middle marker in the window. Thus estimating $\hat{\alpha }$ was the most important step in constructing the LD map.

The model was also fitted at the chromosome level. For each chromosome, we took a random sample of 10, 000 *r*^2^ from all marker pairs to fit the model to obtain an estimate of $\hat{\alpha }$, then computes the mean, ${\hat{\alpha }}_{p,k}$ for chromosome *p* in breed *k*, of 1000 replicates of $\hat{\alpha }$.

## Results

### Genome-wide pattern of LD and persistence of phase

#### Average LD between adjacent SNP pairs

For Duroc, the average distance between adjacent markers is around 40 kb in median (83 kb in mean); the average *r*^2^ is 0.55, the proportion of *r*^2^ > 0.3 is 63%, and of *r*^2^ > 0.2 is 70%. For Landrace, the average distance is around 37 kb in median (72 kb in mean); the average *r*^2^ is 0.50, the proportion of *r*^2^ > 0.3 is 0.58, and of *r*^2^ > 0.2 is 0.67. For Yorkshire, the average distance is around 37 kb in median (72 kb in mean); the average *r*^2^ is 0.50, and the proportion of *r*^2^ > 0.3 is 0.58, and of *r*^2^ > 0.2 is 0.67.

#### Average LD decay over physical distance

**Figure 1:**
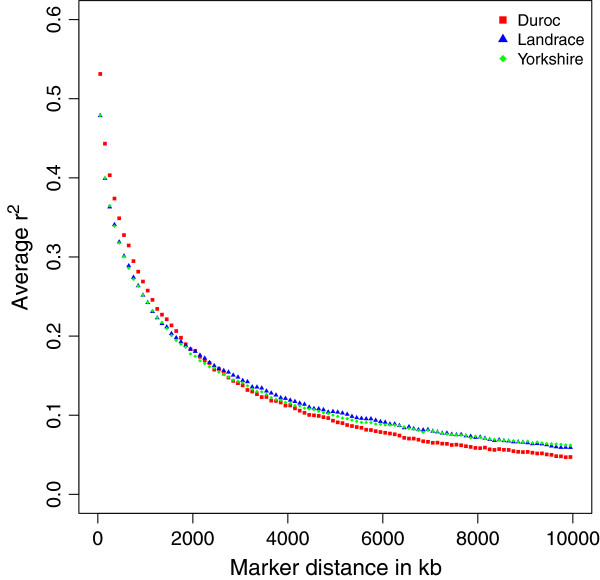
**Decay of average *r***^***2 ***^**over distance for three breeds.** The figure shows the decay of average *r*^2^ for SNP pairs grouped by distance in intervals of 100 kb long covering 0 to 10 Mb across genome.

Figure 1 shows that the average LD decreases as distance between markers increases for all autosomal chromosomes. Comparing different breeds, LD was highest for Duroc at short marker distances, but lowest at long distances; at about 2 Mb, the extent of LD for three breeds dropped to the same level. The curves of LD for Landrace and Yorkshire showed slight differences across the genome.

From Table 1 we observed the average LD also differed between chromosomes. Generally, Chromosome 11 had the least LD across 3 breeds; Chromosome 10 and 12 had relatively low LD compared to other chromosomes. When we compared the patterns of average LD decay over distance for each chromosome, we found that some chromosomes had bigger discrepancy between breeds, while others had little differences between breeds.

**Table 1:** Average linkage disequilibrium (LD) in different autosomal chromosomes for SNPs at various distances

	**Duroc**			**Landrace**			**Yorkshire**		
**Distance**	**0.5 Mb**	**1 Mb**	**5 Mb**	**0.5 Mb**	**1 Mb**	**5 Mb**	**0.5 Mb**	**1 Mb**	**5 Mb**
Chromsome									
1	0.40	0.33	0.14	0.35	0.30	0.14	0.41	0.36	0.17
2	0.41	0.31	0.11	0.30	0.24	0.10	0.35	0.29	0.15
3	0.32	0.25	0.07	0.31	0.22	0.08	0.27	0.22	0.09
4	0.30	0.22	0.08	0.32	0.24	0.09	0.31	0.24	0.07
5	0.28	0.21	0.05	0.24	0.18	0.06	0.28	0.21	0.07
6	0.39	0.30	0.12	0.35	0.27	0.10	0.32	0.26	0.11
7	0.38	0.29	0.09	0.30	0.24	0.07	0.26	0.19	0.07
8	0.38	0.28	0.11	0.29	0.23	0.10	0.34	0.25	0.10
9	0.34	0.26	0.09	0.31	0.23	0.09	0.31	0.26	0.10
10	0.28	0.18	0.04	0.25	0.18	0.04	0.26	0.18	0.04
11	0.26	0.17	0.08	0.26	0.17	0.05	0.23	0.16	0.06
12	0.27	0.19	0.05	0.24	0.18	0.06	0.24	0.16	0.04
13	0.37	0.31	0.11	0.34	0.28	0.15	0.41	0.34	0.16
14	0.39	0.33	0.13	0.41	0.34	0.19	0.35	0.29	0.12
15	0.34	0.27	0.09	0.28	0.22	0.10	0.28	0.21	0.07
16	0.32	0.26	0.07	0.38	0.32	0.13	0.30	0.24	0.08
17	0.31	0.22	0.05	0.33	0.26	0.06	0.27	0.19	0.05
18	0.36	0.23	0.06	0.26	0.21	0.08	0.25	0.15	0.06
All	0.35	0.27	0.09	0.32	0.25	0.10	0.32	0.25	0.10

#### Persistence of phase

For markers with pairwise distances up to 10 kb, we observed persistence of phase to be 0.61 for Duroc-Landrace, 0.57 for Duroc-Yorkshire, and 0.66 for Landrace-Yorkshire, when only considering common SNP across three breeds (Scenario I). When more SNPs were included in the pairwise breed comparisons (Scenario II), persistence of phase decreased slightly to 0.59 for Duroc-Landrace and increased to 0.61 for Duroc-Yorkshire, and 0.70 for Landrace-Yorkshire.

**Figure 2:**
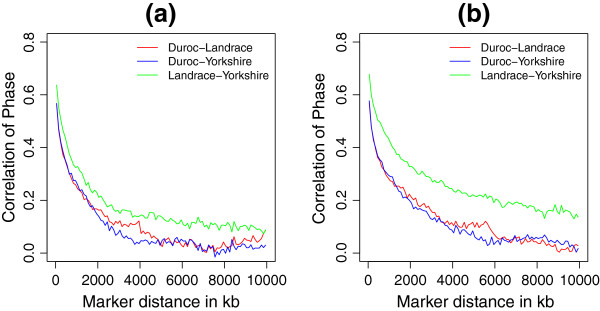
**Correlation of gametic phase compared across breeds over distance.** The figures show correlation of phase between breeds for SNP pairs grouped by distance in intervals of 100 kb long covering 0 to 10 Mb across the genome, **(a)** under Scenario I only included common SNPs for all three breeds, and **(b)** under Scenario II included common SNPs between pair breeds which were not included in the third breed.

For both scenarios, the persistence of phase for Landrace-Yorkshire was higher than that for Duroc-Landrace and Duroc-Yorkshire at different distances (Figure 2). Another common feature of two scenarios was the decrease of persistence of phase with increase of distance. However, the curves in Scenario I fell faster than those in Scenario II, since in Scenario I some SNP pairs specialized in two populations with similar levels of LD were not included. For example, when estimating the persistence of phase for Duroc-Yorkshire, there were about 4000 common SNPs for these two breeds in Scenario II, but not in Scenario I. These extra 4000 pairwise common SNPs captured more consistence in phase of LD between Duroc and Yorkshire. The common SNPs only between Landrace and Yorkshire also increased persistence of phase to higher level in Scenario II, and extended to even longer distances, compared to Scenario I(see Figure 2).

### Local LD and persistence of phase

#### Local variation of ***α*** and LD map

**Figure 3:**
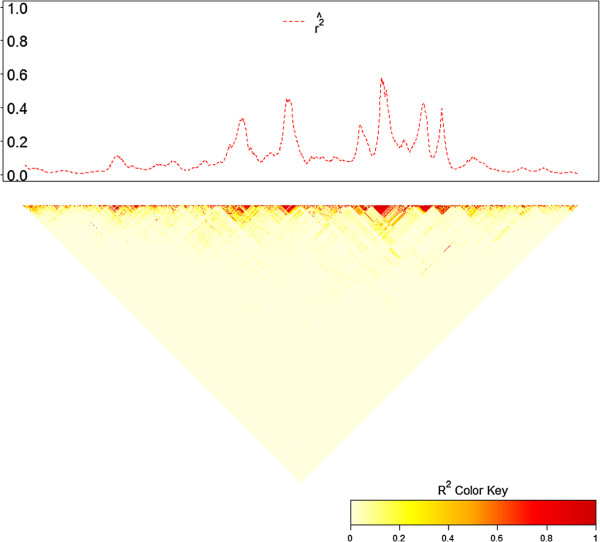
**Comparison of the local LD map with heatmap for Chromosome 1, Duroc.** The upper plot shows how expected *r*^2^, estimated by substituting $\hat{\alpha }$ and 10 Mb in Equation 4, varies along chromosome. Sliding window size *N* = 100. The lower plot is a triangular heatmap, which presents higher LD in darker graphical color blocks.

**Figure 4:**
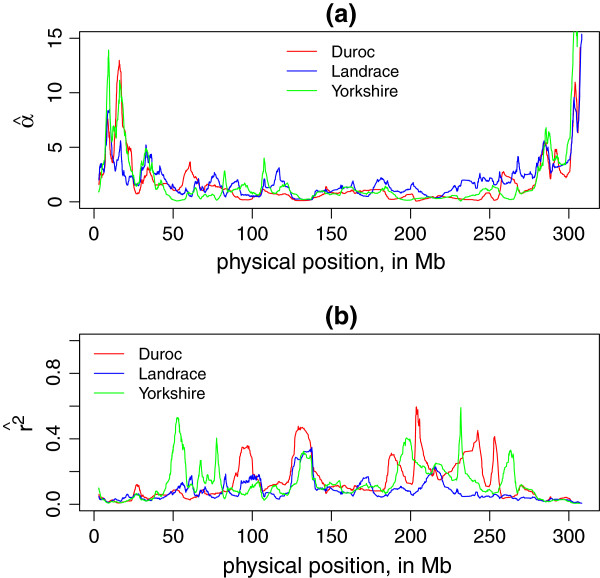
**Local LD decay rate (**$\hat{\alpha }$**) and LD (*r***^***2***^**) along Chromosome 1 for three breeds. (a)** is $\hat{\alpha }$ for each sliding window plotted against the physical position (in Mb) of the middle point SNP in this window, and **(b)** is the expected $\hat{r^{2}}$ estimated by $\hat{\alpha }$ and *d* = 10 Mb plotted against middle point SNP position (in Mb). The sliding window size *N* = 100.

Figure 3 compares our LD map of expected *r*^2^ for Duroc Chromosome 1 with a triangular heatmap [19]: it is shown that the position of the peaks in our plot overlapped with high LD blocks in the heatmap. To visualize the variation in LD along a chromosome, we also plot $\hat{\alpha }$ and expected *r*^2^ against physical position. The fitted value of $\hat{\alpha }$ was substituted back in Equation 4 and a moderate distance 10 Mb (average level of sliding window length, *N* = 100) was used to replace *d*. For example, Figure 4 showed the variation of $\hat{\alpha }$ and expected *r*^2^ along Chromosome 1 for three breeds (window size *N*=100). Here we take physical position of the middle point marker as x-axis.

**Figure 5:**
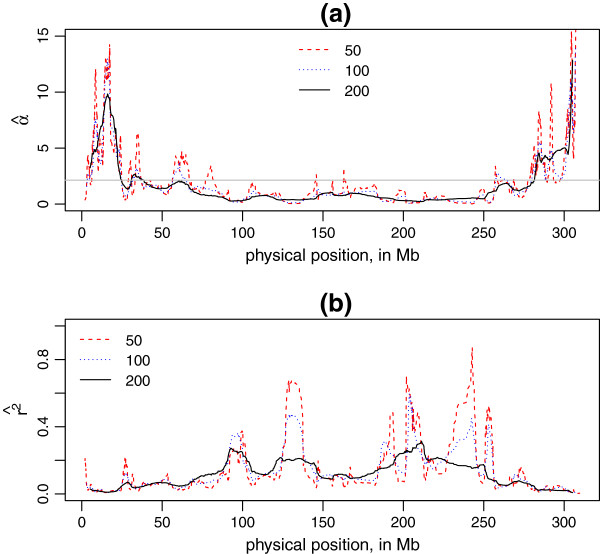
**Local LD decay rate (**$\hat{\alpha }$**) and LD (*r***^***2***^**) in different window sizes.** The figures show effects of different window sizes on the estimates of $\hat{\alpha }$ and expected *r*^2^, illustrated with Chromosome 1, Duroc. **(a)** is a plot of $\hat{\alpha }$ for each sliding window against the physical position (in Mb) of the middle point SNP in this window, and **(b)** is a plot of the expected $\hat{r^{2}}$ by $\hat{\alpha }$ and *d* = 10 Mb against middle point SNP position (in Mb). The window sizes are 50, 100, 200. The grey horizontal line represents the mean $\hat{\alpha }$ for whole Chromosome 1.

We observed that the window size *N* had effects on the estimates of *α* to some extent. Figure 5 indicated that increasing the window size *N* tended to smooth the values of *α*. There was not a clear solution of which window size should be applied. We return to this issue in the discussion.

#### Estimate average ***α*** for each chromosome

**Figure 6:**
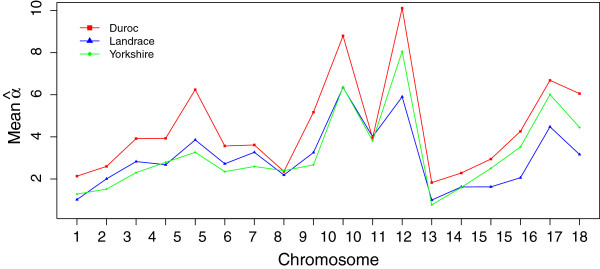
**Estimates of mean(**$\hat{\alpha }$**) for each chromosome and breed.** For each chromosome, a random sample of 10,000 *r*^2^ are fitted in Equation 4 to estimate $\hat{\alpha }$. Repeat this for 1000 replicates and take the mean of 1000 $\hat{\alpha }$, as shown in the plot.

Table 2 contains the mean and standard deviation for 1000 estimates of *α* from sampling 10,000 pairs of *r*^2^ and *d* on each chromosome in three breeds. The range of mean($\hat{\alpha }$) was (1.83, 10.11) for Duroc, (1.00, 5.90) for Landrace, and (0.77, 8.06) for Yorkshire. We found that Duroc had largest $\hat{\alpha }$ compared to the other two breeds for all autosomal chromosomes. Within a breed, Chromosome 10 and 12 had high values of $\hat{\alpha }$, whilst for Chromosome 1 and 13 $\hat{\alpha }$ were relatively small. This was observed for all three breeds. Figure 6 shows that the patterns of mean($\hat{\alpha }$) are similar for the three breeds.

**Table 2:** **The estimates of **$\hat{\alpha }$** for different chromosomes and breeds, mean(**$\hat{\alpha }$**) is the average value of 1000 replicates, and s.d.(**$\hat{\alpha }$**) is the standard deviation**

	**Duroc**		**Landrace**		**Yorkshire**	
**Chromosome**	**mean(**$\hat{\alpha }$**)**	**s.d.(**$\hat{\alpha }$**)**	**mean(**$\hat{\alpha }$**)**	**s.d.(**$\hat{\alpha }$**)**	**mean(**$\hat{\alpha }$**)**	**s.d.(**$\hat{\alpha }$**)**
1	2.14	0.06	1.02	0.02	1.28	0.02
2	2.60	0.06	2.01	0.04	1.52	0.03
3	3.92	0.09	2.83	0.05	2.30	0.04
4	3.93	0.10	2.68	0.05	2.79	0.06
5	6.24	0.19	3.86	0.07	3.27	0.08
6	3.57	0.09	2.72	0.05	2.35	0.05
7	3.61	0.12	3.27	0.08	2.59	0.06
8	2.36	0.05	2.19	0.05	2.40	0.05
9	5.17	0.12	3.26	0.06	2.67	0.06
10	8.79	0.29	6.35	0.12	6.36	0.13
11	3.95	0.13	4.01	0.09	3.82	0.08
12	10.11	0.23	5.90	0.12	8.06	0.17
13	1.83	0.04	1.00	0.02	0.77	0.01
14	2.28	0.04	1.62	0.03	1.60	0.03
15	2.95	0.06	1.63	0.04	2.51	0.05
16	4.26	0.09	2.06	0.04	3.52	0.08
17	6.68	0.13	4.48	0.07	6.01	0.10
18	6.05	0.12	3.16	0.05	4.45	0.07

#### Local persistence of phase

**Figure 7:**
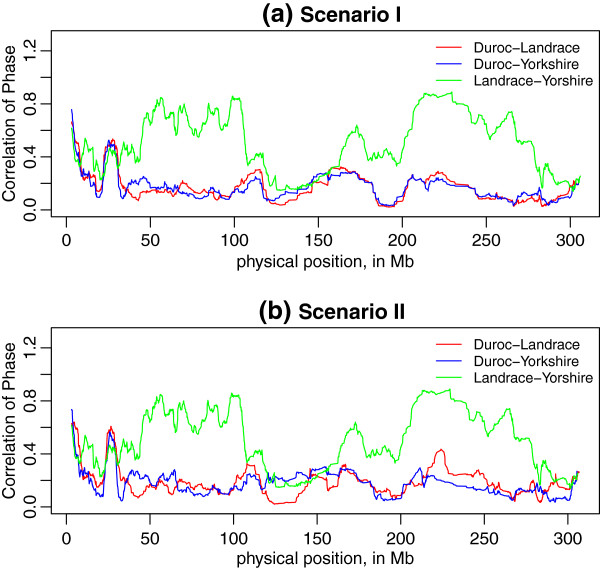
**Persistence of phase at local level for 3 breeds on Chromosome 1 in two scenarios.** The figures show how correlation of phase between breeds varies for SNP pairs in each sliding window, **(a)** under Scenario I which only included common markers for all three breeds, and **(b)** under Scenario II included common markers between pair breeds which were not included in the third breed.

**Figure 8:**
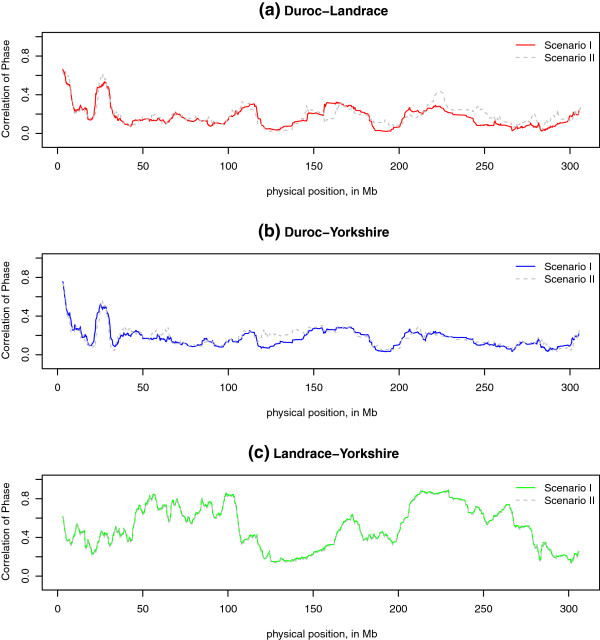
**Comparison of the local persistence of phase between two scenarios for each pair of breeds on Chromosome 1.** The figures show the difference in the correlation of phase calculated by different common SNP sets: solid lines under Scenario I only included common SNPs for all three breeds, and grey dashed lines under Scenario II included common markers between pair breeds which were not included in the third breed.

Local persistence of phase was calculated as the correlation of *r* in each sliding window between two breeds, and plotted against the physical position of the middle point marker in the window. Figure 7 displays the pattern of persistence of phase estimated based on each sliding window: here a window size of *N* = 50 is used. On one hand, compared with Figure 2, we found the maximum value of persistence of phase on local level was 0.95, much higher than genome-wide level, which was only 0.66 for Landrace-Yorkshire with markers distance < 10 kb. Although such a comparison is not precise, it provides insight into the extent of local variation in persistence of phase. On the other hand, the pattern displayed in Figure 7 confirms the closer relationship between Landrace and Yorkshire rather than Duroc with Landrace/Yorkshire even on local level. We also compared local persistence of phase in two scenarios, shown in Figure 8. For Landrace-Yorkshire, the results are almost identical in (c), while for Duroc with Landrace/Yorkshire, at some parts there was discrepancy between two scenarios. Moreover, from the plots of local persistence of phase, we can locate the regions with large difference between breed pairs. For example, in the regions of 30 ∼ 100 Mb and 170 ∼ 260 Mb on Chromosome 1, persistence of phase for Landrace-Yorkshire was much higher than the other two pairs than other regions on the same chromosome (Figure 7). Further, by comparing two scenarios, we found in some regions persistence of phase was affected by the markers excluded in Scenario I. In Figure 7, on regions of 110 ∼ 130 Mb and 210 ∼ 230 Mb, persistence of phase for Duroc with Landrace or Yorkshire increased to a high level in Scenario II from a rather low level in Scenario I.

## Discussion

### Comparison of average LD decay over physical distance across breeds

This study provides an overview of LD patterns against physical distance in three Danish pig breeds, and a comparison between breeds using a 60K SNP panel. Among three Danish breeds, the average LD between adjacent SNPs was greater for Duroc (0.55 in mean, and 0.39 in standard deviation) than Landrace and Yorkshire (0.50 in mean for both, standard deviation is 0.39 for Landrace and 0.38 for Yorkshire). By t-test, we found the difference in the average LD between adjacent markers is significant (P ≪ 0.001) between Duroc with the other two breeds, but not significant between Landrace and Yorkshire (P = 0.19). As we observed in Figure 1, LD over short distance was highest for Duroc, and the difference with the other two breeds was significant at less than 1.8 Mb. LD over short distances indicated “old inbreeding” for Duroc in many generations ago.

Compared with other studies on pig populations, the extent of LD against physical distance (Figure 1) for Danish breeds has similar pattern as US pig breeds [3], insofar as Duroc has highest LD at short distance. However, at long distance Danish Duroc has the lowest LD while US Yorkshire has the lowest LD. There are also differences between our results and other studies, for reasons that are unclear. For example, *r*^2^ decreased to 0.10-0.11 for markers spaced at 5 Mb for Landrace and Yorkshire in our study, while to 0.05-0.06 for US Landrace and Yorkshire [3]. These differences may be partly due to the different sample design. Our sample contains half sibs in each breed, which can attain long distance LD, whereas the US study used trios with less related animals. Moreover, other issues like different levels of inbreeding or selection forces can also result in the discrepancy between LD extent in different countries. But the advantage of our sample is the large sample size, which ensures that the estimates of LD are close to the population values.

### Persistence of phase on genome-wide and local level

Persistence of phase can be used to infer the relatedness of breeds within a species, as well as the reliability of genomic prediction across breeds [2]. In present study, both the genome-wide and local persistence of phase indicated a closer relationship between Landrace and Yorkshire and a more distant relationship between Duroc and Landrace/Yorkshire (Figure 2, Figure 7). This is in agreement with the breeding history of Landrace and Yorkshire, since these two breeds were mixed around 1890 and the herdbook decided to keep them apart soon later. We found that persistence of phase in Danish pig breeds is much lower than other US breeds. US pig breeds [3] showed persistence of phase ranged between 0.87 for Duroc-Yorkshire and 0.92 for Landrace-Yorkshire for markers < 10 kb apart. The reasons for these differences are unclear to us.

The difference in variation explained by the markers which were excluded in Scenario I but included in Scenario II is noteworthy. From Figure 8 we found including more markers in Scenario II increased the extent of persistence of phase between Duroc with Landrace and Yorkshire, compared with which in Scenario I; in contrast, there was no difference in the persistence of phase for Landrace-Yorkshire in both scenarios on some chromosomes. This suggests that the persistence of phase for Duroc with Landrace and Yorkshire is underestimated when only the common markers for the three breeds are included. In addition, the local persistence of phase may be sensitive to the design of SNP chip panel.

### Variation of LD on local and chromosomal level

The purpose of constructing a parametric model of LD as a function of physical distance is to provide a tool to visualize local LD variation along chromosomes, as an LD map, and to allow comparisons of LD across breeds and across genome regions. Here we have demonstrated how the LD map may be used to show variability in LD between different genomic regions and breeds. For example, Figure 4(b) shows average local LD along Chromosome 1 for three breeds (window size *N* = 100): such comparisons would be difficult to make using triangular heatmaps.

It should be noted that at the fine scale, highly complex patterns of allelic association are observed [16,20]. The method proposed here does not aim for optimal prediction of *r*^2^ between specific marker pairs, but intended to aid visualization of patterns of LD along chromosomes, that is, as LD maps. It models local inter-marker LD using a parameter *α* that reflects the local LD decay rate. Patterns of LD along chromosomes can either be shown indirectly using *α* as in Figure 4(a), or more directly by transforming *α* to E(*r*^2^) for a fixed distance as in Figure 4(b). The latter are easier to interpret but involve an arbitrary choice of inter-marker distance.

We chose to use a fixed number of markers in each window, rather than fixing the physical length of the window, in order to keep the same sample size (number of *r*^2^ and *d*) for estimating $\hat{\alpha }$ constant across different windows. We also found that the estimates of $\hat{\alpha }$ were affected by sliding window size, since the fluctuation of $\hat{\alpha }$ decreased when the window size *N* increased, and tended to the mean($\hat{\alpha }$) for a chromosome (horizonal line in Figure 5(a)).

Note that $\hat{\alpha }$ represents a combination of local recombination rate and local effective population size. Since we lack suitable family data to estimate local recombination rates, we cannot disentangle these two parameters without making some assumptions. If we assume that the populations being investigated are under no selection and that the effective population sizes are constant then it may be possible to interpret $\hat{\alpha }$ as a measure of recombination rate both locally and globally. On the other hand, if we assume that recombination rates vary along the chromosomes in the same way across the breeds, then $\hat{\alpha }$ can be interpreted as a local and global measure of effective population size that can be compared across breeds. One advantage of having a parametric model is that it allows us to compare different parameter settings in a statistical framework, e.g. lead to a better fit or easier representation of multi-loci *r*^2^. It allows us to use external data in the model obtained from previous analysis, such as estimate of local recombination rates, and assess whether these lead to a better fit. As a global measure, the patterns of mean ($\hat{\alpha }$) for each chromosome and breed shown in Figure 6 suggest that Duroc has a smaller effective population size on average across all chromosomes. On the other hand, Figure 4(a) also shows for each breed how $\hat{\alpha }$ vary along the chromosome, which may point to genomic regions undergoing selection in each of the breeds. If the curves for local $\hat{\alpha }$ follow the same pattern across breeds, this suggests that this may be due to differences in effective population size. Further investigations are required to better understand the $\hat{\alpha }$ parameter.

Our results are consistent with other researches: the pattern of recombination rates along chromosomes reported in pigs [10] is similar to the pattern of our $\hat{\alpha }$ estimates: higher at the end of chromosomes, and lower in the middle of chromosomes. It has also been found that Chromosome 10 and 12 have the highest average recombination rate, whereas Chromosome 1 and 13 have the lowest [21]. These results are consistent with Table 2, where mean ($\hat{\alpha }$) for Chromosome 10 and 12 are larger than other chromosomes, and that for Chromosome 1 and 13 are smallest. We also found that the estimates of $\hat{\alpha }$ were robust for different samples: when we repeated the analysis in the bigger sample with all genotyped animals, which contains more related individuals, we obtained very similar estimates of $\hat{\alpha }$.

## Conclusion

In our study of LD in three Danish pig breeds, we found that at the genome-wide level, Duroc had the highest LD at short distances, but the lowest LD at long distances, which implied “old inbreeding” for Duroc. Landrace and Yorkshire had similar levels of LD. Persistence of phase was highest between Landrace and Yorkshire, which confirmed the old mixture and closer relationship between these two breeds. The method to estimate and visualize local LD and persistence of phase provided insight into local variation along chromosomes across different breeds. At the chromosomal level, the three breeds showed similar patterns of LD.

## Competing interests

The authors declare that they have no competing interests.

## Authors’ contributions

LW carried out the data analysis and method development, and drafted the manuscript. DE contributed to the method development. DE, PS, LJ and TO contributed to discussion of data analysis and editing the manuscript. TO prepared the genotype data for this study. All authors read and approved the final manuscript.
